# Assessment of Early Growth Response 1 in Tumor Suppression of Esophageal Squamous Cell Carcinoma

**DOI:** 10.3390/jcm11195792

**Published:** 2022-09-29

**Authors:** Yen-Chiang Tseng, Chih-Wen Shu, Hui-Min Chang, Yi-Hsuan Lin, Yen-Han Tseng, Han-Shui Hsu, Yih-Gang Goan, Ching-Jiunn Tseng

**Affiliations:** 1 Division of Thoracic Surgery, Department of Surgery, Kaohsiung Veterans General Hospital, Kaohsiung 813414, Taiwan; 2 Institute of Clinical Medicine, National Yang Ming Chiao Tung University, Taipei 112, Taiwan; 3 Division of Thoracic Surgery, Department of Surgery, Taipei Veterans General Hospital, Taipei 112, Taiwan; 4 Institute of Biopharmaceutical Sciences, National Sun Yat-Sen University, Kaohsiung 80424, Taiwan; 5 Department of Biomedical Science and Environmental Biology, Kaohsiung Medical University, Kaohsiung 80708, Taiwan; 6 Department of Medical Education and Research, Kaohsiung Veterans General Hospital, Kaohsiung 813414, Taiwan; 7 Department of Family Medicine, School of Medicine, National Yang Ming Chiao Tung University, Taipei 112, Taiwan; 8 Department of Public Health, College of Public Health, National Taiwan University, Taipei 100, Taiwan; 9 Department of Chest Medicine, Taipei Veterans General Hospital, Taipei 112, Taiwan; 10 School of Medicine, National Yang Ming Chiao Tung University, Taipei 112, Taiwan; 11 Institute of Emergency and Critical Care Medicine, National Yang Ming Chiao Tung University, Taipei 112, Taiwan

**Keywords:** EGR-1, tumor suppression, esophageal carcinoma

## Abstract

*Background*: Esophageal squamous cell carcinoma (ESCC) is associated with poor survival despite surgical resection, and its pathogenesis has been broadly investigated in the past decade. Early growth response 1 (EGR-1) could involve regulating tumor development in ESCC cells. *Methods*: An attempt was made to examine the molecular and cellular influence of EGR-1 in esophageal cancer cells by RNA extraction, real-time PCR (qRT-PCR), cell culture, small interfering RNA (siRNA) knockdown, western blot, migration assay, and cell viability assay. One hundred and forty-four samples of ESCC were collected from our hospital and analyzed. Significantly higher EGR-1 expression was noted in tumor-adjacent normal tissue compared with tumor lesions. *Results*: The univariate analysis showed no significant impacts of EGR-1 expression on patients’ survival. However, after adjusting for the pathological stage, patients with EGR-1 expression > 68th percentile had lower risks of cancer-related death. Moreover, knockdown of EGR-1 significantly enhanced cell migration, invasion, and resistance to chemotherapeutic agents in two ESCC cell lines. *Conclusions*: EGR-1 plays a key role in tumor suppression involving tumor viability suppression and reflects the treatment effect of current chemotherapy for ESCC.

## 1. Introduction

Squamous cell carcinoma of the esophagus, or esophageal cancer, is the eighth-most common malignancy in the world, with incidence rates varying by more than 21-fold among different regions [1,2]. It is associated with poor survival despite surgical resection. Many clinicopathological variables, including the depth of tumor invasion, lymph node involvement, lymphovascular invasion, intramural metastasis, and the stage of the disease, have been assessed to predict the prognosis. Multiple molecular changes have also been investigated to elucidate the mechanism of esophageal squamous cell carcinoma (ESCC) tumorigenesis [3]. 

Early growth response 1 (EGR-1), or the early growth response-1 gene product, is a zinc-finger transcription factor of 59,000 Daltons. It appears to activate transcription by binding to DNA as a monomer. It regulates cell growth and differentiation in response to signals, such as mitogens, growth factors, and stress stimuli [4]. Analysis of specific human tumor cells and tissues indicates that EGR-1 acts as both a tumor suppressor and a tumor promoter [5]. EGR-1 is commonly suppressed in gliomas in human glioma independent of *p16/INK4a/ARF* and *Mdm2*. The suppression is less crucial in tumors bearing *p53* mutations. These results implicated an EGR-1 growth regulatory mechanism as a target of inactivation during tumor progression [6]. EGR-1 is also decreased or undetectable in small-cell lung and human breast tumors [7,8,9].

The complexity of the molecular pathway involved in tumorigenesis of esophageal cancer has been reported by Hsu et.al. [8]. The relationship between EGR-1 expression and eukaryotic translation initiation factor 4E-binding protein 1 (4E-BP1) gene expression in esophageal cancer cells has been investigated [10]. The EGR-1 expression was reported to increase in TE2 esophageal cancer (TE2) cells compared with other esophageal cell lines, and knockdown of EGR-1 could increase 4E-BP1 expression in TE2 cells, which became sensitive to rapamycin treatment [10]. Although EGR-1 is a crucial transcription factor in controlling cell growth, proliferation, differentiation, and angiogenesis, its role in the development of esophageal cancer is poorly understood despite the high frequency of this disease in many parts of the world. Wu et al. (2004) found that ESCC patients with EGR-1 overexpressed in their cancer tissue and who underwent radiotherapy had a better prognosis [11]. This study reported that radiotherapy up-regulated EGR-1 expression and that it may be a potential radiation response marker of ESCC.

Immunohistochemistry showed that EGR-1 is overexpressed in 80% of esophageal tumor tissues, as described in the study of Wang’s group [11,12]. They investigated the relationship between the EGR-1 and esophageal cancer cells. Esophageal squamous carcinoma WHCO1 cells stably transfected with EGR-1 short hairpin RNA displayed a 55% reduction in EGR-1 protein levels, 50% reduction in cell proliferation, a 50% reduction in cyclin-dependent kinase 4 (CDK4) levels, and a 2-fold induction in p27Kip1 levels associated with a G2-M cell cycle arrest. EGR-1-related signaling in esophageal cancer may present potential target molecules for therapeutic intervention. Peng et al. (2010) reported that knockdown of EGR-1 with its siRNA could overcome the protective effect of hypoxic conditions and increase the sensitivity of tumor cells to vinblastine treatment [13]. Increased ESCC cell growth and invasion ability by EGR-1 knockdown were also reported in vitro experiments [14].

In this study, EGR-1 was hypothesized to play a vital role in tumor suppression in ESCC cells. Tumors and adjacent normal cells from patients with ESCC were investigated to explore the EGR-1 expression and clinical outcome involving ESCC pathogenesis. In addition, gene silencing with scrambled siRNA or siRNA against EGR-1 was employed to inspect the role of EGR-1 in cancer cell migration, invasion, and cell viability in response to chemotherapeutic agents in ESCC cells. 

## 2. Materials and Methods

### 2.1. Sample Collection

The samples of ESCC were collected from 144 patients diagnosed with ESCC between 2002–2018 at Kaohsiung Veterans General Hospital, Taiwan. None of the patients received chemotherapy or radiotherapy before acquiring the tumor specimen. Patient demographic and clinical data were also collected from medical records, including sex, cell differentiation, pathological stage, Tumor, Node, Metastasis (TNM) classification, tumor subsites, and tumor recurrence time. Pathologic TNM classification was determined according to the guidelines of the 2002 American Joint Committee on Cancer (AJCC) system. The Institutional Review Board at our hospital approved this study to comply with the Declaration of Helsinki.

### 2.2. Real-Time PCR (qRT-PCR)

Total RNA was extracted with illustra triplePrep Kit (GE Healthcare, Chicago, IL, USA, 28-9425-44). A total of 1 μg RNA was reverse-transcribed with SuperScriptIIl RNase Reverse Transcriptase (Invitrogen, Lot no 1992043) for cDNA synthesis. The qPCR reaction was carried out using SYBR Green Master Mix (Applied Biosystems, 4385612) in a StepOnePlus^TM^ system (Applied Biosystems). GADPH was used as an internal control. The relative mRNA expression was calculated using the 2^−ΔΔCq^ method and normalized by GADPH. The primer sequences are listed in Supplemental Table S1. 

### 2.3. Determination of Pathological TNM Classification

Pathological TNM classification has been determined at the time of the initial resection of the tumor following the guideline of the 2002 American Joint Committee on Cancer (AJCC) system.

### 2.4. Cell Culture

Esophageal cancer cell lines CE48T and CE81Twere obtained from Dr. Cheng-Po Hu at Taipei Veterans General Hospital and cultured in Dulbecco’s modified Eagle’s medium (DMEM) (Invitrogen-Gibco, Carlsbad, CA, USA), with 10% heat-inactivated fetal bovine serum (Biological Industries, Kibbutz Beit-Haemek, Israel), 100 U/mL penicillin (Invitrogen-Gibco, Carlsbad, CA, USA), 1% MEM non-essential amino acids (NEAA), and 100 μg/mL streptomycin (Invitrogen-Gibco, Carlsbad, USA) at 37℃ in a humidified 5% CO_2_ atmosphere. Cells were grown in Corning tissue-culture-treated plastic (Corning, Inc., Corning, NY, USA).

### 2.5. siRNA Knockdown

The siRNA-mediated EGR-1 knockdown was carried out in the esophageal cancer cell line to observe the cell growth and viability of ESCC tumor cells. The sequences of four different EGR-1 siRNA oligos pools (Supplemental Table S1) were synthesized by Dharmacon, Inc. All transient transfections of the siRNA against EGR-1 (siEGR-1) oligos pool at a final concentration of 10 nM were accomplished with Lipofectamine RNAiMAX Transfection Reagent (Invitrogen, Carlsbad, CA, USA) by following the manufacturer’s protocols. Esophageal cancer cells of 3 × 10^5^ per well were seeded into six-well flat-bottom plates containing 1 mL medium. The siRNA oligonucleotides and RNAiMAX Transfection Reagent were separately diluted by 100 μL of the opti-MEM medium, and then the mixture was incubated for 15–20 min. The cells were incubated with the transfection medium and incubated overnight at 37 °C in a humidified atmosphere of 5% CO_2_. Cells were incubated for 24, 48, and 72 h before harvesting. Non-silencing control (NSC) was used at the same concentration of siRNAs.

### 2.6. Western Blot Analysis

The cells were briefly rinsed in PBS and lysed with RIPA buffer (1% NP-40, 50 mM Tris-Cl pH 7.5, 150 mM NaCl, 0.25% sodium deoxycholate 1% SDS, and a protease inhibitor cocktail). The cell lysates were resolved by 10–12% SDS-PAGE and transferred electrophoretically onto nitrocellulose membranes. The membranes were blocked with bovine serum albumin (BSA) for the anti-phosphoserine antibody and with 0.1% casein in 0.2× PBS for the other antibodies. The membranes were incubated with the primary antibodies, including anti-EGR1 (#4153, cell signaling), at 4 °C overnight. The proteins were developed with HRP-labeled secondary antibody and detected with ECL reagent. The membrane was scanned and analyzed for protein expression levels with LI-COR^®^ Odyssey^®^ Imaging System (LI-COR, Inc.).

### 2.7. Migration Assay

The functional outcome after EGR-1 knockdown was assessed using migration and invasion assays. Transwell invasion and migration assays were performed migration wound-healing assay using 8-μm pore inserts (Greiner Bio-One, Stroud, UK). In the invasion assay, the cells were knocked down with siRNA for 48 h and then seeded at a concentration of 1.5 × 10^5^ cells into the top chamber of transwell plates coated with 0.5% Matrigel in 300 μL of DMEM containing 1% FBS. To the bottom, wells were added as a complete medium to stimulate invasion. After seeding for 24 h, the cells were fixed and stained with 0.1% crystal violet. The cells that had invaded through the Matrigel and had reached the reverse side were imaged under a microscope at a magnification of 200× and were quantified with ImageJ. For the invasion assay, the cells were knocked down with siRNA for 72 h and then were seeded at a concentration of 2 × 10^5^ cells into an inset chamber by a complete medium of 70 µL. After planting for 24 h, sterile forceps were used to remove the plastic inserts. The cell patch was washed with 1 mL of fresh culture medium. The wound-healing width was then viewed under the microscope at 200× magnification.

### 2.8. Cell Viability Assay

Esophageal cancer cells were seeded into 96-well flat-bottom plates of 5 × 10^3^ cells per well containing 100 µL of the medium. The siRNA oligonucleotides and Lipofectamine RNAiMAX reagent were separately diluted by 25 µL of the Opti-MEM medium, and then the mixture was incubated for 15–20 min. The cells were incubated with the transfection medium for 48 h at 37 °C in a humidified atmosphere of 5% CO_2_. 

Next, various concentrations of Cisplatin (2019 Merck KGaA, Darmstadt, Germany and/or its affiliates, CAS-NO. 15663-27-1), 5′FU (2019 Merck KGaA, Darmstadt, Germany and/or its affiliates, CAS-NO. 51-21-8), VP16 (CAS-NO. 33419-42-0), and paclitaxel (2019 Merck KGaA, Darmstadt, Germany and/or its affiliates, CAS-NO. 33069-62-4) were separately diluted in 20 µL of the medium. Then, cells were incubated with the drug medium and treated for 24, 48 h at 37 °C in a humidified atmosphere of 5% CO_2_. The cell viability was determined using manufacturer’s instructions according to CellTiterGlo Luminescent kit. All experiments were performed in triplicate.

### 2.9. Statistical Analysis

Baseline characteristics are presented by the mean, standard deviation, median, and 25th and 75th quartile for continuous and n for categorical variables. The comparison of gene expression profiles between tumor part and adjacent normal in two pairs of ESCC cancer patients was performed by Wilcoxon signed-rank test due to the paired data without normal distribution. EGR-1 expression between the characteristics was compared by the Wilcoxon rank sum test or Kruskal–Wallis test due to small sample sizes, whereas categorical data were analyzed using Fisher’s exact test. Cox regression models were used to estimate the hazard ratio (HR) and 95% confidence intervals (CIs) of the risk of cancer-related death. However, adjusting survival curves was also conducted based on the Kaplan–Meier method. All statistical analysis was carried out by SAS (V.9.4; SAS Institute, Cary, NC, USA).

## 3. Results

The gene expression profiles between tumor part and adjacent normal in two pairs of ESCC cancer patients in the esophageal cancer tissue bank of Kaohsiung Veterans General Hospital and The Cancer Genome Atlas (TCGA) were obtained. All the samples of gene expression were analyzed by using qRT-PCR. The EGR-1 expression was significantly higher in tumor-adjacent normal tissue than in tumor lesions from our tissue bank (5.87 ± 10.22 vs. 3.97 ± 7.58, *p* = 0.0015, Table 1). The EGR-1 expression was also higher in tumor-adjacent normal tissue than in tumor lesions from the TCGA database (Table 2). The EGR-1 presentation on tumor lesions at 25th, 50th, and 75th percentile was 0.52, 1.30, and 3.45, respectively. The EGR-1 expression cut-off point for cancer-related death was 2.45 (68th percentile), calculated by the Youden using ROC curve analysis.

Baseline characteristics and the corresponding EGR-1 expression level are presented in Table 3. None of the patients received neoadjuvant therapy. The results indicated no association between the various clinical profiles and EGR-1 expression, including when expression of EGR-1 was classified into low and high expression groups cut off at the 68th percentile of EGR-1 expression.

The cancer-related survival rates between different EGR-1 expression groups are listed in Table 4. The univariate analysis showed no significant impacts of EGR-1 expression on patients’ survival in each EGR-1 expression group model. However, after adjusting AJCC pathological stage, patients with EGR-1 expression higher than 75th and 68th percentile had significantly lower risks of cancer-related death (75th: HR: 0.45 95% CI: 0.20~0.98, *p* = 0.045; 68th: HR: 0.43 95% CI: 0.20~0.94, *p* = 0.034). The results showed patients with higher EGR-1 expression had a better survival rate (Supplemental Figure S1). 

**Figure 1:**
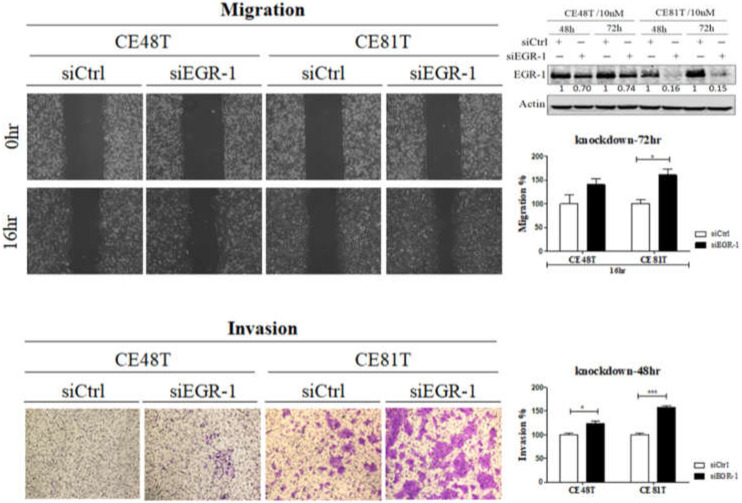
Effects of EGR-1 knockdown on migration and invasion ability in esophageal cancer cell lines CE48T and CE81T after transfected with scramble siRNA (siCtrl) or siRNA against EGR-1 (siEGR-1). The migration ability of ESCC cells in representative image (left panel) and quantitated in the right panel. For invasion assay, cells were stained with crystal violet (left panel) and quantitative on the right panel. (* = *p* < 0.05, *** = *p* < 0.001).

After the EGR-1 gene knockdown, the initial outcome showed an effective gene knockdown in different cancer cell lines, including CE48T and CE81T (Figure 1). The migration was significantly increased in siEGR-1 CE81T cells. The invasion was increased dramatically in the siEGR-1 group in both ESCC cell lines compared to the respective siCtrl (Figure 1).

**Figure 2:**
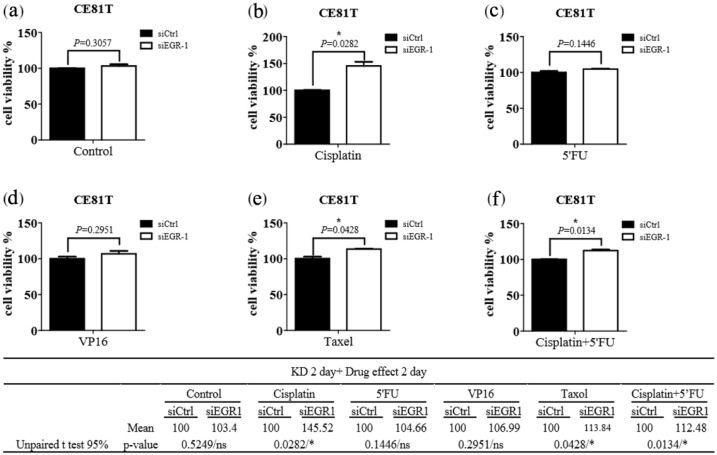
Efficacy of chemotherapeutic agents on the viability of CE81T cells. CE81T harboring scramble siRNA (siCtrl) or siRNA against EGR-1 (siEGR-1) for 48 h were exposed to (**a**) Control, (**b**) Cisplatin (10 µM), (**c**) 5′FU (20 µM), (**d**) VP16 (10 µM), (**e**) Taxol (250 nM), and (**f**) Cisplatin (10 µM) + 5′FU (20 µM) for 48 h. The cell viability was determined with CellTiter Glo as described in the methods section. The results were obtained from three independent experiments and expressed as mean ± SEM. * *p* < 0.05.

**Figure 3:**
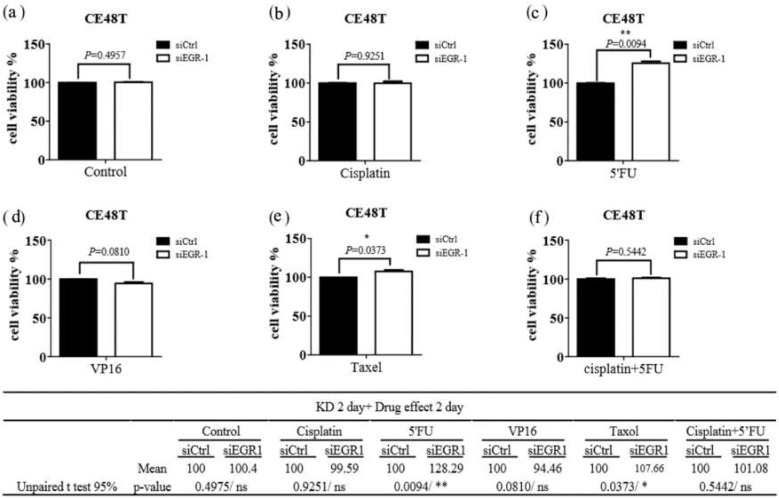
Efficacy of chemotherapeutic agents on CE48T cells. CE48T harboring scramble siRNA (siCtrl) or siRNA against EGR-1 (siEGR-1) for 48 h were exposed to (**a**) Control, (**b**) Cisplatin (10 µM), (**c**) 5′FU (20 µM), (**d**) VP16 (10 µM), (**e**) Taxol (250 nM), and (**f**) Cisplatin (10 µM) + 5′FU (20 µM) for 48 h. The cell viability was determined with CellTiter Glo as described in the methods section. The results were obtained from three independent experiments and expressed as mean ± SEM. * *p* < 0.05, ** *p* < 0.01.

The functional change in drug resistance in ESCC cells after EGR-1 gene knockdown was also assessed. The cell viability assays were applied for evaluation using siEGR-1 groups compared with the siControl group after chemotherapy, including cisplatin [15], 5-FU [16], and etoposide (VP16) [17], taxol [18], and cisplatin with 5-FU [19]. Knockdown of EGR-1 significantly increased the resistance of cisplatin or/and taxol in CE81T cells (Figure 2); the concentration of these anticancer drugs used in cells was referenced from the following literature [15,16,17,18,19,20,21], while silencing EGR-1 significantly increased resistance of 5′FU and taxol in CE48T cells (Figure 3). Therefore, EGR-1 presented the function of tumor viability suppression and reflected better treatment effects of current chemotherapy for ESCC (Figure 2 and Figure 3). In addition, the expected outcome of the siControl group compared with the siEGR-1 group was noted.

## 4. Discussion

The present study revealed a vital role of EGR-1 in ESCC patients from the tissue bank of Kaohsiung Veterans General Hospital and the TCGA database. Both evaluations indicated that EGR-1 involves tumor suppression in ESCC. In the two ESCC cell line models, the enhanced invasion, migration, and resistance against chemotherapeutic reagents in the EGR-1 gene knockdown group compared to control also indicated the tumor suppressive effect of EGR-1. Both clinical and in vitro findings concluded that EGR-1 plays a crucial role in tumor suppression of ESCC.

EGR-1 regulates cell growth and differentiation in response to signals, such as mitogens, growth factors, and stress stimuli [4]. Some results of the previous papers are contrary to ours, indicating that EGR-1 is an oncogene in many types of cancer [8,22,23,24]. In ovarian cancer, EGR-1 mediated epidermal growth-factor-induced downregulation of E-cadherin expression via Slug in ovarian cancer cells. Loss of the cell adhesion protein E-cadherin increases the invasive capability of ovarian cancer cells [22]. In prostate cancer, EGR-1 expression was significantly increased in tumors with Gleason scores of 8–10 [23]. The NAB2 (NGF-1A binding protein), which represses the transcriptional activity of EGR-1, is down-regulated in prostate carcinomas. Upregulation of EGR-1 and loss of its repressor NAB2 contribute to increasing levels of EGR-1 activity in prostate cancer [25]. Li et al. indicated that EGR-1 induced angiogenic and osteoclastogenic factor expression, leading to prostate cancer metastasis [26]. In breast cancer, after transfection with siRNA-EGR-1, the cell growth and capacity of two Human breast carcinoma cells, SK-BR3 and MCF-7, were lower than the control group [24]. However, some research supports our hypothesis that EGR-1 may be a tumor suppressor in many types of cancer. A previous study analysis of specific human tumor cells and tissues indicated that EGR-1 participates in tumor suppression [5]. Another study reported that EGR-1 upregulation and nuclear translocalization could be regulated by 2′-Benzoyloxycinnamaldehyde (BCA), activating proapoptotic target genes and inducing prostate cancer cell death [27]. Yet another paper demonstrated that the apoptosis-stimulating protein of p53 forms an inter-regulatory loop with early growth response 1 (EGR-1) and promotes apoptosis via inhibiting cytoprotective autophagy, independent of the well-documented p53-dependent mechanisms [28]. The experimental results of this article confirm our hypothesis and provide a possible tumor suppressor mechanism of EGR-1. Although not many articles discuss the role of human EGR-1 in tumor suppressor genes, we have noticed many articles discussing that the homologous gene Egr1 in mice can inhibit the growth of various tumors [29,30]. Based on our clinical investigation and in vitro study, we believe that EGR-1 plays a double-edged sword role in cancer development. In our clinical study, the qRT-PCR data indicated that EGR-1 was predominantly overexpressed in tumor-adjacent normal tissue parts. This result suggests that EGR-1 surrounds the tumor like a guard to prevent tumor cells invade to other organs. Interestingly, our in vitro study supports our hypothesis. The data demonstrated that the knockdown of EGR-1 by the siRNA technique significantly promotes the migration and invasion ability of ESCC cell lines. This finding leads us to believe that EGR-1 may play a role as a tumor suppressor of ESCC.

This study also focused on the predictive value of EGR-1 with chemo-sensitivity. A previous study demonstrated that reducing the levels of EGR-1 caused TNBC cells to become more resistant to PTX [31]. In vitro, EGR-1 levels were decreased by transfecting parental MDA-MB-231 cells with 5 nM of an EGR-1 siRNA (compared to a control siRNA, siCtrl) for 28 h before the addition of a two-fold PTX titration series from 78–0.6 nM, or a media-only control. The results indicated that cells receiving siEGR-1 were more resistant to PTX than ctrl-transfected cells. Another interesting study demonstrated that downregulation of EGR-1 gene expression sensitized radioresistant cells to IR [32]. These studies support our study in that inhibition of EGR1 expression promotes cancer cell sensitivity to drugs. Together, these results suggest that patients with different cancers can take some medicines or natural compounds to induce EGR-1 expression before chemotherapy to promote cancer cell sensitivity to medication.

There are several limitations to this study. Compared to the tumor samples, less than four tumor-adjacent standard samples were obtained from the TCGA database, which might cause bias in the statistical analysis due to the small sample numbers and unbalance between groups. Although the migration and invasion ability of tumor cells was increased after the EGR-1 knockdown in this study, the experimental data are still insufficient to conclude that EGR-1 is a tumor suppressor in ESSC. This study only investigated the effect of siEGR-1 in esophageal cancer. The overexpression of EGR-1 should be further verified to make the results more convincing. Furthermore, this article refers to the observed phenomenon without a complete mechanism study.

In conclusion, this study investigated the effect and significance of EGR-1 in ESCC patients. The qRT-PCR data revealed that EGR-1 is predominantly higher-expressed in tumor-adjacent normal tissues compared to tumor tissues. The siRNA experiment results indicated that the knockdown of EGR-1 significantly increases the migration and invasion ability of ESCC cell lines. Furthermore, the cell viability assay demonstrated that higher EGR-1 expression increases the sensitivity of ESCC to chemotherapeutics. Based on our findings, we will use the EGR-1 cut-off value to evaluate our subsequent clinical patients further. Together, these results suggest EGR-1 may function as a tumor suppressor gene in ESCC.

## Figures and Tables

**Table 1:** The comparisons of EGR-1 expression ESCC and corresponding tumor-adjacent normal tissues.

Variables	Tumor-Adjacent Normal (n = 144)		Tumor (n = 144)		*p* Value *
	Mean ± SD	Median (Q1~Q3)	Mean ± SD	Median (Q1~Q3)	
EGR-1	5.87 ± 10.22	2.12 (0.93–7.14)	3.97 ± 7.58	1.30 (0.52–3.45)	**0.0015**

EGR-1, early growth response 1; ESCC, squamous cell carcinoma of the esophagus; SD, standard deviation; Q1, 25th percentile; Q3, 75th percentile. * *p* value estimated by Wilcoxon signed-rank test. The statistically significant *p* value is in bold.

**Table 2:** The comparisons of EGR1 expression in the tissues of ESCC using TCGA database.

Variables	Tumor-Adjacent Normal (n = 3)		Tumor (n = 93)		Metastatic (n = 1)		*p* Value *
	Mean ± SD	Median	Mean ± SD	Median	Mean ± SD	Median	
EGR1	14.04 ± 0.69	14.04	12.30 ± 0.13	12.27	11.56	-	**0.021**

EGR1, early growth response 1; TCGA, The Cancer Genome Atlas; ESCC, esophageal squamous cell carcinoma; SD, standard deviation. * *p* value estimated by Kruskal–Wallis one-way ANOVA test. The statistically significant *p* value is in bold.

**Table 3:** Clinical pathologic outcomes in patients with ESCC.

Variables		No (%)	EGR-1 (Continuous)		EGR-1 > 68th Percentile		
			Median (Q1~Q3)	*p*-Value	Yes (n = 46)	No (n = 98)	*p*-Value ^c^
Sex				0.70 ^a^			0.65
	Male	139 (96.5)	1.26 (0.51~3.69)		95 (96.94)	44 (95.65)	
	Female	5 (3.5)	2.42(1.33~2.61)		3 (3.06)	2 (4.35)	
Cell Differentiation				0.16 ^b^			0.16
	Well	1 (0.7)	6.37		0 (0)	1 (2.17)	
	Moderate	108 (75.0)	1.52 (0.55~3.86)		71 (72.45)	37 (80.43)	
	Poor	35 (24.3)	1.05 (0.37~1.86)		27 (27.55)	8 (17.39)	
AJCC pathological				0.84 ^b^			0.68
	I	15 (10.4)	0.72 (0.52~7.18)		9 (9.18)	6 (13.04)	
	II	72 (50.0)	1.21 (0.53~2.66)		52 (53.06)	20 (43.48)	
	III	53 (36.8)	1.71 (0.51~4.03)		34 (34.69)	19 (41.3)	
	IV	4 (2.8)	1.41 (0.66~16.39)		3 (3.06)	1 (2.17)	
T classification				0.71 ^b^			0.81
	T1	15 (10.4)	0.91 (0.50~2.53)		11 (11.22)	4 (8.7)	
	T2	30 (20.8)	1.63 (0.61~3.04)		20 (20.41)	10 (21.74)	
	T3	96 (66.7)	1.19 (0.50~3.97)		64 (65.31)	32 (69.57)	
	T4	3 (2.1)	1.26 (0.13~1.97)		3 (3.06)	0 (0)	
N classification				0.47 ^b^			0.66
	N0	69 (47.9)	1.15 (0.54~2.71)		50 (51.02)	19 (41.3)	
	N1	66 (45.8)	1.68 (0.49~4.03)		42 (42.86)	24 (52.17)	
	N2	8 (5.6)	1.15 (0.49~4.32)		5 (5.1)	3 (6.52)	
	N3	1 (0.7)	0.17		1 (1.02)	0 (0)	
M classification				0.93 ^a^			1.00
M0		140 (97.2)	1.30 (0.52~3.45)		95 (96.94)	45 (97.83)	
M1		4 (2.8)	1.41(0.66~16.39)		3 (3.06)	1 (2.17)	

EGR-1, early growth response 1; ESCC, esophageal squamous cell carcinoma; AJCC, American Joint Committee on Cancer. ^a^ Wilcoxon rank sum test; ^b^ Kruskal–Wallis test; ^c^ Fisher’s exact test.

**Table 4:** Univariate and adjusted hazard ratio of EGR-1 expression for patients’ survival.

Models ^a^	Univariate Analysis		Multivariate Analysis ^b^	
	HR (95% CI)	*p*-Value	aHR (95% CI)	*p*-Value
EGR-1 (Continuous)	0.97 (0.93~1.01)	0.178	**0.96 (0.92~0.99)**	**0.024**
EGR-1 > 25th percentile	0.60 (0.29~1.23)	0.163	0.59 (0.29~1.22)	0.155
EGR-1 > 50th percentile	0.95 (0.53~1.70)	0.867	0.76 (0.41~1.39)	0.372
EGR-1 > 75th percentile	0.67 (0.33~1.36)	0.267	**0.45 (0.20~0.98)**	**0.045**
EGR-1 > 68th percentile (ROC)	0.63 (0.31~1.27)	0.193	**0.43 (0.20~0.94)**	**0.034**

^a^ Model was separated and conducted. ^b^ with AJCC pathological stage. EGR-1, early growth response 1; AJCC, American Joint Committee on Cancer. Significant *p* values (*p* < 0.05) are in bold.

## Data Availability

The datasets generated during the current study are available from the corresponding author on reasonable request.

## Supplementary Materials

The following supporting information can be downloaded at: https://www.mdpi.com/article/10.3390/jcm11195792/s1, Figure S1: Impact of EGR-1 expression levels on survival by in patients with ESCC; Table S1: The sequences used in real-time PCR and siRNA-mediated EGR-1 knockdown.
