# Supplementary material for: Assessment of Early Growth Response 1 in Tumor Suppression of Esophageal Squamous Cell Carcinoma
Source: J Clin Med. 2022 Sep 29;11(19):5792. doi: 10.3390/jcm11195792 (PMC9572560; doi:10.3390/jcm11195792)

Supplemental Table S1. The sequences used in real-time PCR and siRNA-mediated EGR-1 knockdown.

Item	Sequence
EGR-1 forward	5'-GGTCAGTGGCCTAGTGAGC-3'
EGR-1 reverse	5'-GTGCCGCTGAGTAAATGGGA-3'
GAPDH forward	5'-TGCACCACCAACTGCTTAGC-3'
GAPDH reverse	5'- GGCATGGACTGTGGTCATGAG-3'
EGR-1 siRNA oligo 1	5'-GAUGAACGCAAGAGGCAUA-3'
EGR-1 siRNA oligo 2	5'- CGACAGCAGUCCCAUUUAC-3'
EGR-1 siRNA oligo 3	5'-GGACAUGACAGCAACCUUU-3'
EGR-1 siRNA oligo 4	5'- GACCUGAAGGCCCUCAAUA-3'

Abbreviation: EGR-1, Early growth response1; GAPDH, Glyceraldehyde 3-phosphate dehydrogenase. siRNA, Small interfering RNA.

Supplemental Figure S1. Impact of EGR-1 expression levels on survival by in patients with ESCC.

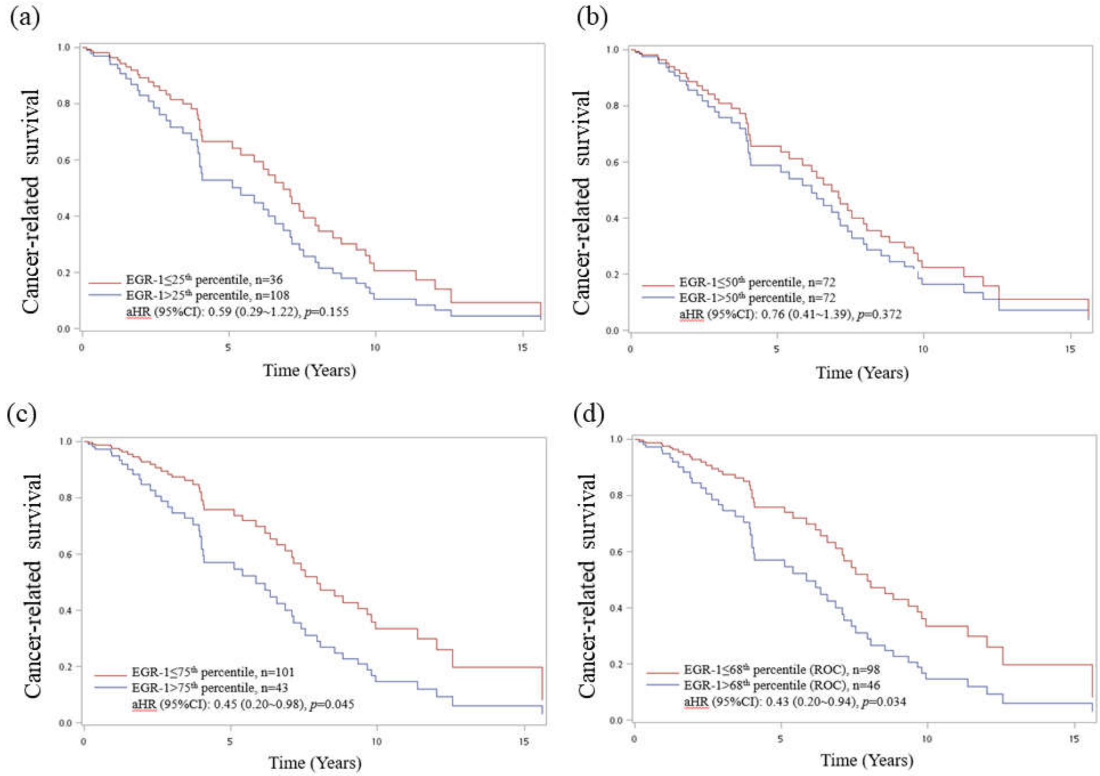

Supplement: Supplementary file 1 [file jcm-11-05792-s001.zip › jcm-1893356-supplementary.pdf]
